# miRNA as Potential Biomarkers of Breast Cancer in the Lebanese Population and in Young Women: A Pilot Study

**DOI:** 10.1371/journal.pone.0107566

**Published:** 2014-09-18

**Authors:** Farah J. Nassar, Maya El Sabban, Nathalie K. Zgheib, Arafat Tfayli, Fouad Boulos, Mark Jabbour, Nagi S. E. l. Saghir, Rabih Talhouk, Ali Bazarbachi, George A. Calin, Rihab Nasr

**Affiliations:** 1 Department of Biology, Faculty of Arts and Sciences, American University of Beirut, Beirut, Lebanon; 2 Department of Anatomy, Cell Biology and Physiological Sciences, Faculty of Medicine, American University of Beirut, Beirut, Lebanon; 3 Department of Pharmacology, Faculty of Medicine, American University of Beirut, Beirut, Lebanon; 4 Department of Internal Medicine, Faculty of Medicine, American University of Beirut, Beirut, Lebanon; 5 Department of Pathology, Faculty of Medicine, American University of Beirut, Beirut, Lebanon; 6 Department of Experimental Therapeutics, Division of Cancer Medicine, University of Texas MD Anderson Cancer Center, Houston, Texas, United States of America; University of Connecticut Health Center, United States of America

## Abstract

Relative to western populations, the percentage of women diagnosed with breast cancer at a young age in Lebanon is high. While the younger age of the Lebanese population compared to the West certainly contributes to this difference, potential genetic, reproductive and/or biological factors likely play an important role. The objective of this study is to investigate the contribution of miRNAs in this setting through the analysis of the expression of five reported dysregulated miRNAs, miR-148b, miR-10b, miR-21, miR-221, and miR-155 in 20 normal and 57 cancerous breast tissues from Lebanese breast cancer patients. After finding their relative expression by quantitative reverse transcription real time PCR, the results were analyzed with respect to the patients’ clinical and histopathology presentations. Compared to normal breast tissues, significant upregulation of miR-155, miR-21 and miR-148b, notable downregulation of miR-10b and non-significant expression of miR-221 were observed in tumor tissues. Moreover, miR-10b was significantly underexpressed in estrogen/progesterone receptor (ER/PR) negative tumors relative to ER/PR positive tumor tissues. miR-155 was also significantly overexpressed in postmenopausal patients and in those of age at diagnosis greater than 40 years old as well as in PR negative or in human epidermal growth factor 2 (Her2) positive tissues. This study is the first one to report miRNA expression patterns in Lebanese breast cancer patients. We found that differential miRNA expression in breast cancer could be variable between Lebanese and Western populations. miR-10b was positively correlated with the ER and PR status and miR-155 could be a noteworthy biomarker for the menopausal state, age at diagnosis, PR and Her2 status. Hence, miRNA can be used as biomarkers for early breast cancer detection.

## Introduction

Breast cancer is one of the leading health concerns worldwide, affecting over one million women every year. In Lebanon, it is one of the most common type of cancer constituting about one third of all female cancers [1]. Interestingly, a significant number of Lebanese breast cancer patients were noted to be of young age at the time of diagnosis as 22% of the cases were below the age of 40 years old compared to 6% in the Western populations [2]. Moreover, Lebanese women who are diagnosed at young age (less than 35 years old) and in their premenopausal state were shown to present with a more aggressive disease and poorer survival in spite of adequate therapy [3]. The presence of signs of more aggressive features in breast cancer in young women, and the occurrence of breast cancer in young Lebanese women 10 years earlier in age than those in the West, strengthens the importance of determining the biological factors behind those differences, and perhaps revealing novel biomarkers for early screening and detection of breast cancer.

Since the discovery of microRNA (miRNA) in *C. elegans* twenty years ago, this major subclass of non-coding RNA molecules act as gene modulators mostly at the posttranscriptional level by causing translation repression or degradation of mRNA [4]. miRNAs play diverse roles in normal cellular processes such as cell cycle, proliferation and apoptosis as well as in disease conditions including cancer, diabetes, neuro-degenerative disorder and cardiac hypertrophy [5], [6]. miRNA was first correlated with breast cancer by Iorio and his colleagues. Using microarray analysis, they discovered a differential miRNA profile pattern between cancerous and normal breast tissues [7]. miRNAs were later shown to modulate tumor suppressor and oncogenic pathways thereby contributing to the different stages of breast cancer and acting as regulators of cell cycle progression, apoptosis, angiogenesis, epithelial to mesenchymal transition, tumor microenvironment, migration, invasion and metastasis [8]. Previous studies attempted to correlate the different miRNA expression profiles in tumor tissues with the histological diagnosis stage and other variables [9]. Interestingly, studies have also shown that miRNAs are not only present in tissues, but also in biological fluids including plasma. This suggests that miRNAs have the potential to serve as non-invasive diagnostic and prognostic biomarkers as well as possible therapeutic targets.

The oncogenic role of miR-155, miR-21, miR-10b and miR-221 and their potential use as biomarkers in breast cancer have been reported extensively [10]–[13]. In addition, circulating miR-148b was found to have a discriminatory potential for young breast cancer patients [14]. This study aims to investigate the expression patterns of these miRNAs, which are known to be dysregulated in breast cancer, in Lebanese breast cancer patients of varying clinical and histopathology presentations.

## Materials and Methods

### Breast cancer tissue specimens

After obtaining the approval of the Institutional Review Board (IRB) at the American University of Beirut, formalin fixed paraffin embedded (FFPE) sections from invasive ductal carcinoma specimens (N = 57) and normal adjacent tissues (NAT) (N = 20) were obtained at the American University of Beirut Medical Center (AUBMC) in Lebanon from samples that were collected between 1997 and 2012 and were previously recruited for a breast cancer study [15]. Patients who participated in the originally IRB approved study signed a written informed consent form. Those who agreed that their stored breast cancer FFPE tissues, if available, are used for further research were included in this study. The Pathology department at AUBMC provided four to five 20 µm ribbons from the tumor biopsy core and five 20 µm ribbons from the adjacent normal tissue when available. Sections were taken from paraffin embedded tissue blocks containing at least 90% viable invasive tumor cells. The corresponding adjacent normal breast tissue sections included epithelial mammary parenchyma and were entirely free of significant pathology such as atypical hyperplasia or carcinoma. Benign proliferative changes such as adenosis were allowed. Clinical and pathological data, such as the age at diagnosis, menopausal status, tumor pathology, stage, grade, ER status, PR status, and Her2 overexpression was available for the 57 tumor samples.

### Total RNA extraction

Total RNA was extracted from 20 normal and 57 tumor tissues in accordance to the protocol of RecoverAll Total Nucleic Acid Isolation Kit for FFPE samples (Ambion, USA). Briefly, FFPE samples were first deparaffinized using xylene at 50°C and then washed twice with ethanol to remove the xylene. Proteins were digested by incubation of the samples with protease enzyme for 15 min at 50°C and then at 80°C. Total RNA was then captured in glass-fiber filter cartridges with the help of an isolation additive mixture followed by washing steps with high ethanol-wash buffers. After DNA digestion using DNase for 30 min, RNA was purified by washing and elution. RNA concentration and quality was assessed using the Nanodrop ND1000 and then stored at −80°C. Only high quality samples were used for downstream applications.

### miRNA expression by quantitative real time-polymerase chain reaction (RT-qPCR)

Reverse transcription of ten nanograms of the total RNA was performed using the TaqMan MicroRNA Reverse Transcription Kit (Applied Biosystems, USA) according to the manufacturer’s instructions. Small nuclear RNA RNU6B, human hsa-miR-16, hsa-miR-21, hsa-miR-221, hsa-miR-148b, hsa-miR-10b, and hsa-miR-155 primers and probes were purchased as part of the TaqMan microRNA Assays Kit (Applied Biosystems, USA) with validated efficiency. cDNA synthesis was carried out in a multiplex reaction set up whereby two miRNA primers (for example miR-148b and miR-10b) were used in each reaction with the endogenous control, miR-16 or RNU6B. RT-qPCR was performed using BioRad CFX96 Real Time System, C1000 Thermal Cycler (Germany). Reactions using 10 µl of 2x TaqMan Universal Master Mix with no Amperase Uracil N-glycosylase (UNG) (Applied Biosystems, USA), 1 µl of the corresponding 20x microRNA probe, 4 µl of DEPC treated water, and 5 µl of cDNA were performed in duplicates for each miRNA probe. cDNA Synthesis and RT-qPCR were repeated twice for each sample and each plate included: no reverse transcription control (NRT), no template control (NTC) and normal breast tissue samples. The normalization of the tumor tissues was based on the normal tissues present in the RT-qPCR plate to ensure inter-run calibration. The cycling conditions were 95°C for 10 min and 40 cycles of 95°C for 15 seconds and an annealing temperature of 60°C for 60 seconds. Using the ΔΔCt equation, the relative expression of the experimental miRNA was determined in the tumor samples compared to the normal tissue specimens using miR-16 or RNU6B as an endogenous control.

### Statistical Analysis

Statistical Analysis was performed using SPSS software package version 18. Wilcoxon’s rank sum test for one sample was used to compare the miRNA expression in the tumor versus the normal tissues. Mann-Whitney *U* nonparametric test was used for comparing two different groups such as premenopausal and postmenopausal, but if more than two groups were compared, Kruskal-Wallis nonparametric test was used. Spearman’s rho (*r*) was calculated to find a correlation between two variables. A *p*-value<0.05 was considered statistically significant.

## Results

### Baseline Demographics

The clinical and pathological data have been retrieved for all 57 samples of the breast cancer patients (Table 1). The breast cancer pathology was invasive ductal carcinoma for all the cases with 78.9% ER positive, 66.7% PR positive and 33.3% Her2 overexpression (defined as score 3 by IHC or score 2 by IHC with positive FISH) [16]. Most of the tumors were moderately to highly differentiated (42.1% and 47.4% for tumor grade 2 and 3). 52.6% of the tumors were T2 with local growth and an average tumor size between two to five centimeters, 53.8% had lymph node involvement, and most had no distant metastasis (M0: 93%) according to the American Joint Committee on Cancer’s (AJCC) Staging [17].

**Table 1:** Relative expression of miR-148b, miR-10b, miR-21, miR-221 and miR-155 in 57 tumor breast tissues from Lebanese patients of different clinical and histopathology presentations by RT-qPCR with miR-16 used as an endogenous control.

Patient Presentation	Relative Expression^a^ of miRNAs and *p*-Value of different categories^b^										
	N (%)	miR-148b	*p*-Value	miR-10b	*p*-Value	miR-21	*p*-Value	miR-221	*p*-Value	miR-155	*p*-Value
***Age***											
**≤40 years**	13 (22.8)	1.763	0.732	0.532	0.761	4.659	0.123	0.829	0.171	2.349	**0.029**
**>40 years**	44 (77.2)	1.851		0.493		5.512		0.997		2.976	
***Menopausal State***											
**Premenopausal**	30 (52.6)	1.796	0.477	0.534	0.472	4.973	0.523	0.948	0.672	2.543	**0.038**
**Postmenopausal**	27 (47.4)	1.875		0.435		5.54		0.859		3.778	
***ER status***											
**Negative**	12 (21.1)	1.811	0.914	0.26	**0.006**	4.799	0.557	0.902	0.883	4.591	0.096
**Positive**	45 (78.9)	1.828		0.559		5.242		0.933		2.561	
***PR status***											
**Negative**	19 (33.3)	1.717	0.71	0.382	**0.049**	5.169	0.826	1.395	0.416	4.367	**0.025**
**Positive**	38 (66.7)	1.828		0.573		5.223		0.916		2.403	
***Her2 overexpression***											
**Negative**	38 (66.7)	1.826	0.906	0.551	0.66	5.095	0.264	0.978	0.654	2.412	**0.021**
**Positive**	19 (33.3)	1.941		0.403		5.728		0.871		3.686	
***Lymph Node Involvement***											
**No**	24 (46.2)	1.52	0.091	0.484	0.409	6.175	0.452	1.047	0.43	2.573	0.515
**Yes**	28 (53.8)	1.999		0.562		5.113		0.895		2.908	
***Tumor Size***											
**T1: ≤2** **cm**	21 (36.8)	1.823	0.978	0.484	0.913	5.483	0.895	1.102	0.698	2.378	0.051
**T2: >2** **cm but ≤5** **cm**	30 (52.6)	1.834		0.534		5.187		0.865		3.008	
**T3: >5 cm**	4 (7.0)	1.55		0.515		5.145		0.868		4.457	
**T4: any size with direct extension to chest wall and/or to skin**	2 (3.5)	1.661		0.424		5.985		0.889		0.792	
***Tumor Grade***											
**G1: Well differentiated**	6 (10.5)	1.226	0.261	0.54	0.525	3.505	0.171	0.881	0.303	2.169	0.429
**G2: Moderately differentiated**	24 (42.1)	1.849		0.576		5.451		1.051		2.639	
**G3–G4: Poorly differentiated**	27 (47.4)	1.828		0.414		5.242		0.871		3.138	
***Metastasis***											
**No**	53 (93)	1.828		0.536		5.419		0.993		2.585	
**Yes**	4 (7)	2.004		0.276		2.281		0.629		3.047	

a median of relative expression.

b Mann-Whitney *U* and Kruskal-Wallis nonparametric test were used for comparing different groups.

### miRNA expression in breast cancer and NATs

**Figure 1:**
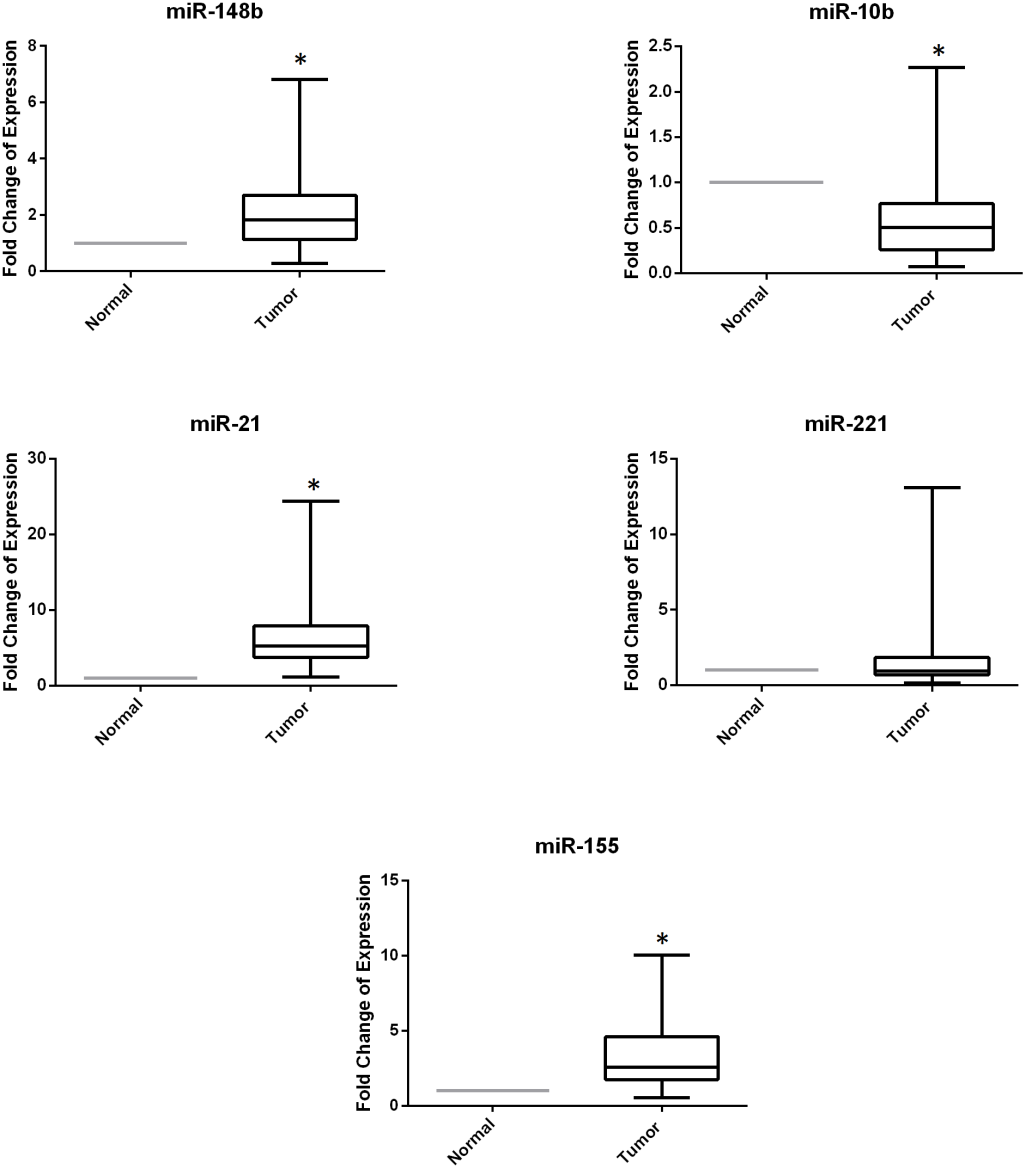
miRNA expression is dysregulated in breast cancer tissues in the Lebanese population. This boxplot displays the fold change of relative expression of miR-148b, miR-10b, miR-21, miR-221 and miR-155 in 57 tumor versus 20 normal adjacent breast cancer tissues that is measured by RT-qPCR with miR-16 used as an endogenous control. Whiskers represent minimum and maximum, the top, the bottom and the band in the box represent the first and third quartile and the median respectively. *denotes *p*<0.05, using Wilcoxon’s signed-rank test.

In order to compare normal and tumor breast tissues in the expression of the selected miRNAs (miR-148b, miR-10b, miR-21, miR-221, and miR-155), reverse transcription real time PCR was performed on cDNA transcribed from RNA that was extracted from 20 normal and 57 tumor breast formalin fixed paraffin embedded tissues. After normalization in the RT-qPCR with miR-16 or RNU6B as endogenous control, Wilcoxon’s rank sum test was used to identify the miRNAs that were significantly different in the normal and tumor samples. Similar results were obtained upon normalizing to either one of the endogenous controls. miR-148b, miR-21 and miR-155 were significantly overexpressed (*p*<0.05) in the tumor tissues versus the normal tissues. While miR-221 expression was not significantly different, miR-10b was found to be significantly underexpressed in tumor as compared to normal tissues (Figure 1). Table 2 also shows the expression of the five miRNA in tumor versus normal breast tissues in sample groups of specific menopausal status (premenopausal or postmenopausal) or of specific age at diagnosis (younger than 40 or older than 40 years old). The expression results of these miRNAs in these groups were the same as when taking the total sample size with miR-148b, miR-21 and miR-155 significantly overexpressed and miR-10b significantly underexpressed in tumor as compared to normal tissues (Table 2).

**Table 2:** Relative expression of miR-148b, miR-10b, miR-21, miR-221 and miR-155 in 57 tumor versus 20 normal breast tissues of Lebanese patients by RT-qPCR with miR-16 used as an endogenous control.

Patient Presentation	Relative Expression^a^ of miRNAs and *p*-Value^b^ in tumor versus normal tissues										
	N (%)	miR-148b	*p*-Value	miR-10b	*p*-Value	miR-21	*p*-Value	miR-221	*p*-Value	miR-155	*p*-Value
***All***	57 (100)	1.828	**0.0001**	0.502	**0.0001**	5.205	**0.0001**	0.933	0.3080	2.585	**0.0001**
**Premenopausal**	30 (52.6)	1.796	**<0.0001**	0.534	**0.0007**	4.973	**<0.0001**	0.948	0.522	2.543	**<0.0001**
**Postmenopausal**	27 (47.4)	1.875	**<0.0001**	0.435	**<0.0001**	5.540	**<0.0001**	0.859	0.496	3.778	**<0.0001**
**Age ≤40 years**	13 (22.8)	1.763	**0.005**	0.532	**0.0002**	4.659	**0.0002**	0.829	0.216	2.349	**0.006**
**Age >40 years**	44 (77.2)	1.851	**<0.0001**	0.493	**<0.0001**	5.512	**<0.0001**	0.997	0.099	2.976	**<0.0001**

a median of relative expression.

b Wilcoxon’s rank sum test was used to compare tumor versus normal breast tissues.

Relative expression of these miRNAs in breast cancer patients of different menopausal status (premenopausal or postmenopausal) or different age at diagnosis (≤ or >40 years) is also shown.

### miRNA expression and menopausal state or age at diagnosis

**Figure 2:**
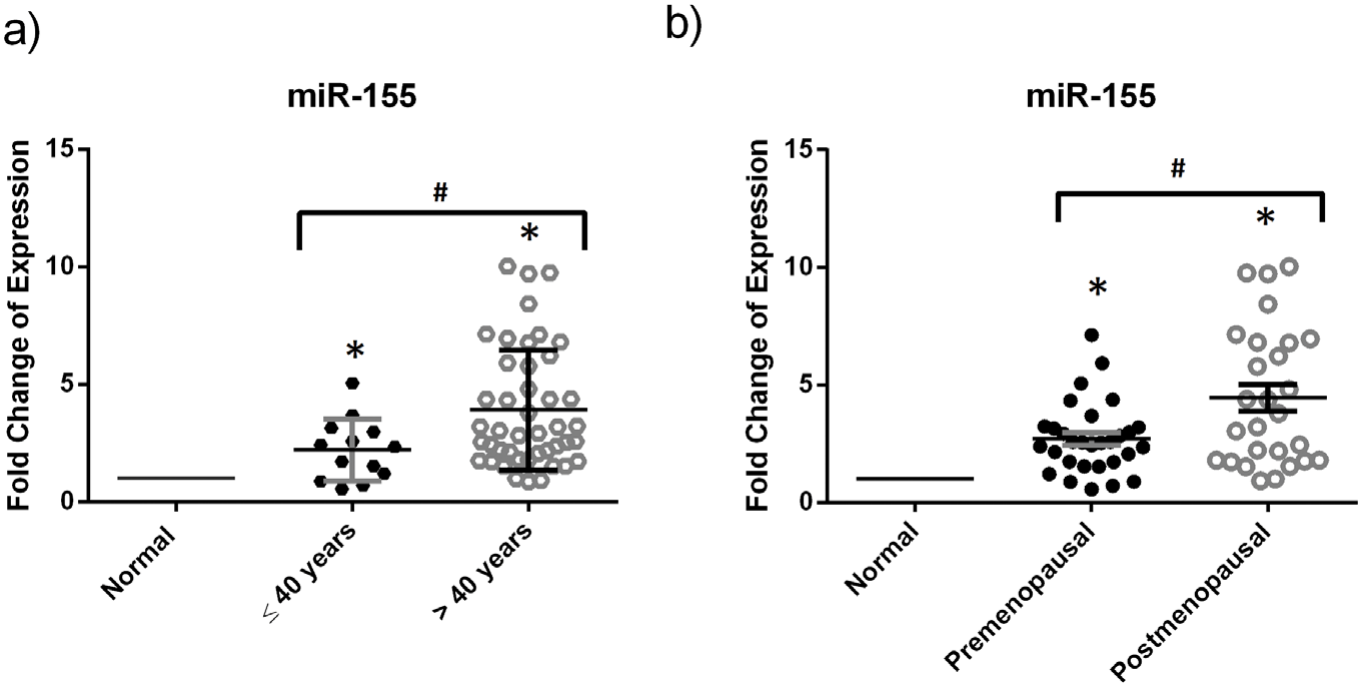
miR-155 is upregulated in breast cancer tissues of postmenopausal women and those greater than 40 years old. This dotplot shows the fold change of the significantly differentially expressed miRNAs upon dividing patients into groups based on the age at diagnosis and menopausal status. Relative expression of miR-155 in breast cancer tissues from patients of a) age at diagnosis less than or equal to 40 years and age greater than 40 years or of b) premenopausal and postmenopausal status was measured by RT-qPCR with miR-16 used as an endogenous control. The plot represents the mean with the standard error of mean as error bars. *denotes *p*<0.05 for tumor versus normal using Wilcoxon’s signed-rank test. #denotes *p*<0.05, using Mann-Whitney *U* test.

The variation in the miRNA expression according to menopausal status and age group (≤40 years old and >40 years) was analyzed by Mann-Whitney *U* test. When looking at the menopausal status or age at diagnosis, no significant difference in the miRNA expression was observed between the premenopausal and postmenopausal groups or between the age groups for miR-21, miR-10b, miR-148 and miR-221. miR-155 was significantly upregulated in postmenopausal patients when compared to premenopausal patients, as well as in the age group greater than 40 years as compared to that of less than or equal to 40 years old (Figure 2).

### miRNA expression and clinical histopathological features

**Figure 3:**
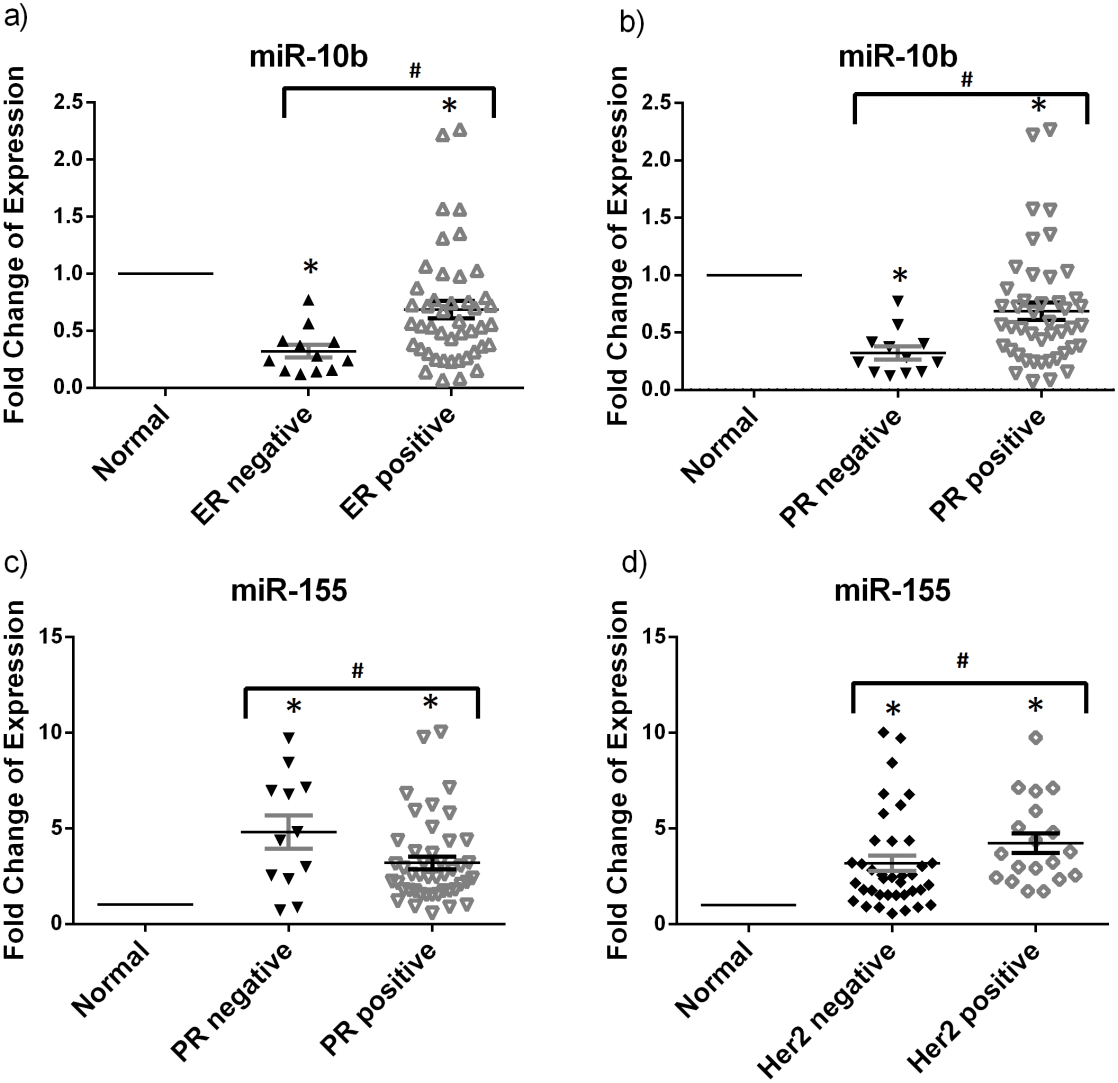
Relation of miR-155 and miR-10b with the receptor profile. This dotplot shows the fold change of the significantly differentially expressed miRNAs upon dividing patients into groups based on ER, PR or Her2 status. Relative expression of a) miR-10b in ER negative and positive breast cancer tissues b) and c) miR-10b and miR-155 in PR negative and positive breast cancer tissues respectively d) miR-155 in Her2 negative and positive breast cancer tissues was measured by RT-qPCR with miR-16 used as an endogenous control. The plot represents the mean with the standard error of mean as error bars. *denotes *p*<0.05 for tumor versus normal using Wilcoxon’s signed-rank test. #denotes *p*<0.05, using Mann-Whitney *U* test.

To evaluate whether certain miRNAs are dysregulated with the various clinical and histopathological features associated with breast cancer, we analyzed our data by nonparametric statistical tests (Mann-Whitney *U* and Kruskal-Wallis tests) after dividing the samples into groups with differential ER or PR expression or Her2 overexpression, lymph node involvement, tumor size and grade. No analysis was performed on the different histological types since all tumor samples were of ductal carcinoma type, the most common type in the Lebanese population. Upon ER grouping, there were significantly lower miR-10 expression levels in ER negative specimens relative to the positive ones (Figure 3a). Upon PR grouping, data revealed a significant upregulation of miR-155 and downregulation of miR-10b in the PR negative samples when compared to the PR positive samples (Figure 3b and c). As for Her2 overexpression grouping, only miR-155 was significantly upregulated in the Her2 overexpressed samples as compared to those not overexpressing Her2 (Figure 3d). Spearman-Rho test was out carried out to find correlations between the miRNA relative expression and the different clinical histopathological features. miR-10b was positively correlated with the ER and PR status with correlation coefficients of *r* = 0.368 and *r* = 0.262 (*p*<0.05 for each) respectively and miR-155 was positively correlated with Her2 expression and negatively correlated with PR expression with correlation coefficients of *r* = 0.308 and *r* = −0.3 (*p*<0.05 for each)respectively. Lymph node involvement, tumor size and grade groups did not reveal any significant difference in the expression of the selected experimental miRNAs (Table 1).

## Discussion

Compared to western populations, Lebanese women are more likely to be diagnosed with breast cancer at a younger age, which has been reported to be associated with adverse pathological features such as histological high grade, negative hormone receptors, HER2 overexpression and advanced stage at presentation and hence carries a poorer prognosis [18]. Recent research explaining breast cancer at young age is focusing on possible molecular aberrations such as RANKL, PI3K pathways in order to identify possible therapeutic targets [19], [20]. miRNAs are now widely investigated in different types of cancers and particularly in breast cancer. Their differential expression may be useful for the study of etiology of breast cancer, prognosis, and possible therapeutic targeting. This study looks at dysregulated miRNA profiles for Lebanese breast cancer patients where a significant number of patients are of young age. In this work, we have studied the expression of specific miRNAs (miR-148b, miR-10b, miR-21, miR-221 and miR-155) by RT-qPCR in cancerous and normal adjacent breast tissues and investigated whether their expression is linked to this increasing frequency for early onset of breast cancer in the Lebanese women. Our data showed that miR-148b, miR-21 and miR-155 were significantly overexpressed in tumor tissues versus normal tissues. While miR-221 expression was not significantly different, miR-10b was also found to have significantly decreased expression in tumor compared to normal tissues (Table 2). The upregulation of miR- 21 and miR- 155 in tumor versus normal tissues was consistent with the literature and their oncogenic role [7]. miR-21 has been reported as an oncomir that contributes to motility invasion and metastasis by targeting tumor suppressors such as phosphatase and tensin homolog (PTEN), tropomyosin 1, programmed cell death 4 (PDCD4) and mammary serine protease inhibitor (Maspin) [21]–[23]. miR-155 has been also shown to be an oncomir acting as apoptosis suppressor and inducer of cell proliferation and chemoresistance through targeting Caspase3, suppressor of cytokine signaling 1 (SOCS1) and Forkhead transcription factor (FOXO3a) tumor suppressors [24], [25]. On the contrary, the upregulation of miR-148b was not in accordance with its reported function as a tumor suppressor that was downregulated in breast tumors [26]. However, our results are in agreement with the increased expression of miR-148b obtained in ovarian carcinoma [27]. Interestingly, miR-148b was also shown to be upregulated in the serum of young-aged breast cancer patients [14]. Downregulation of miR- 10b, an oncomir with metastatic potential, was obtained in metastasis free patients [7], [28] and that is the case with patients in this study who mostly have no metastasis. As for miR-221, no significant difference in its expression was obtained in Lebanese breast cancer patient population which contrasts previous reports that demonstrated its increase in breast cancer due to its oncogenic role by regulating proliferation, angiogenesis and multiple tumor suppressors [10], [29]. Hence, differentially expressed miRNA between normal and tumor breast tissues could be variable between Lebanese and Western populations. Remarkably, it was previously found that there is little overlap between the differentially expressed miRNAs identified from Caucasian American breast cancer patients and those from African American groups, suggesting a potential ethnic difference in miRNAs [30].

The significant downregulation of miR-10b in ER/PR negative samples as compared to ER/PR positive samples and its positive correlation with ER and PR status establishes a relationship between miR-10b and ER and PR expression. In addition, the significant upregulation of miR-155 in PR negative samples compared to PR positive and its negative correlation with PR status were in agreement with a published study by Lu et al. in 2012. Similarly, the significant overexpression of miR-155 in Her2 positive relative to Her2 negative samples and its positive correlation with Her2 status were similar to what was previously reported [31], [32]. miR-155 could not only predict the PR and the Her2 status but also its significant upregulation in postmenopausal samples and in patients of age greater than 40 years old made it a considerable breast cancer biomarker for postmenopausal patients or for those greater than 40 years old. On the other hand, finding differentially expressed miRNA specific for premenopausal patients or for those less than or equal to 40 years would be more beneficial for diagnosis of early onset breast cancer in Lebanese patients. Even though many studies have correlated the miR-21 expression with lymph node involvement and histological grade, our study did not show any statistically significant increase that could be due to the small sample size [33], [34].

It is important to note that the NAT controls used in this study were not accurate normal breast tissues from healthy subjects but were the available normal tissues adjacent to the tumors. Nonetheless, we found very little variation between each of the normal samples that were analyzed, leading us to confirm with greater confidence that the variations between the tumor samples’ miRNA patterns is a result of the tumor characteristics. RT-qPCR analysis was performed depending on the normal tissue run in the same plate with the tumor tissues and not depending on the average normal as some of the probes showed variability between different runs but consistently within the same run. Moreover, even though this study was performed on a small subset of miRNAs with relatively small sample size, it was a hypothesis generating pilot study that has revealed the necessity of global microarray analysis to identify differentially expressed miRNA in the Lebanese breast cancer patient population.

In conclusion, this study is the first one to report miRNA expression profiles in a Lebanese breast cancer population, a population with high incidence of early age-onset breast cancer. miR-10b was positively correlated with the ER and PR status. Moreover, miR-155 was shown as a noteworthy biomarker for the menopausal state, PR and Her2 status (Table 1). This suggests that the underlying causes for the trend of early onset breast cancer in the Lebanese population may be related to biological factors in addition to yet undetermined genetic mutations and relationship to reproductive factors [35]. Accordingly and due to the difference in microRNA expression between different populations and between different ethnic groups, global miRNA dysregulation through microarray profiling could help determine the existence of specific miRNA signatures in the Lebanese breast cancer population, and also to identify miRNA biomarkers for the young and premenopausal patients in general.

## References

[1] El Saghir NS, Khalil MK, Eid T, El Kinge AR, Charafeddine M, et al (2007) Trends in epidemiology and management of breast cancer in developing Arab countries: a literature and registry analysis. Int J Surg 5: 225–233. 17660128 10.1016/j.ijsu.2006.06.015

[2] Shamseddine A, Musallam K (2010) Cancer Epidemiology in Lebanon. Middle East Journal of Cancer 1: 41–44.

[3] El Saghir NS, Seoud M, Khalil MK, Charafeddine M, Salem ZK, et al (2006) Effects of young age at presentation on survival in breast cancer. BMC Cancer 6: 194. 16857060 10.1186/1471-2407-6-194 PMC1555600

[4] Lee RC, Feinbaum RL, Ambros V (1993) The C. elegans heterochronic gene lin-4 encodes small RNAs with antisense complementarity to lin-14. Cell 75: 843–854. 8252621 10.1016/0092-8674(93)90529-y

[5] Ha TY (2011) MicroRNAs in Human Diseases: From Cancer to Cardiovascular Disease. Immune Netw 11: 135–154. 21860607 10.4110/in.2011.11.3.135 PMC3153666

[6] Li Y, Kowdley KV (2012) MicroRNAs in common human diseases. Genomics Proteomics Bioinformatics 10: 246–253. 23200134 10.1016/j.gpb.2012.07.005 PMC3611977

[7] Iorio MV, Ferracin M, Liu CG, Veronese A, Spizzo R, et al (2005) MicroRNA gene expression deregulation in human breast cancer. Cancer Res 65: 7065–7070. 16103053 10.1158/0008-5472.CAN-05-1783

[8] Li L, Xiao B, Tong H, Xie F, Zhang Z, et al (2012) Regulation of breast cancer tumorigenesis and metastasis by miRNAs. Expert Rev Proteomics 9: 615–625. 23256672 10.1586/epr.12.64

[9] Lu J, Getz G, Miska EA, Alvarez-Saavedra E, Lamb J, et al (2005) MicroRNA expression profiles classify human cancers. Nature 435: 834–838. 15944708 10.1038/nature03702

[10] Chen WX, Hu Q, Qiu MT, Zhong SL, Xu JJ, et al (2013) miR-221/222: promising biomarkers for breast cancer. Tumour Biol 34: 1361–1370. 23529451 10.1007/s13277-013-0750-y

[11] Ma L (2010) Role of miR-10b in breast cancer metastasis. Breast Cancer Res 12: 210. 21067538 10.1186/bcr2720 PMC3096969

[12] Mattiske S, Suetani RJ, Neilsen PM, Callen DF (2012) The oncogenic role of miR-155 in breast cancer. Cancer Epidemiol Biomarkers Prev 21: 1236–1243. 22736789 10.1158/1055-9965.EPI-12-0173

[13] Singh R, Mo YY (2013) Role of microRNAs in breast cancer. Cancer Biol Ther 14: 201–212. 23291983 10.4161/cbt.23296 PMC3595302

[14] Cuk K, Zucknick M, Heil J, Madhavan D, Schott S, et al (2013) Circulating microRNAs in plasma as early detection markers for breast cancer. Int J Cancer 132: 1602–1612. 22927033 10.1002/ijc.27799

[15] Zgheib NK, Shamseddine AA, Geryess E, Tfayli A, Bazarbachi A, et al (2013) Genetic polymorphisms of CYP2E1, GST, and NAT2 enzymes are not associated with risk of breast cancer in a sample of Lebanese women. Mutat Res 747–748: 40–47. 10.1016/j.mrfmmm.2013.04.004 23628324

[16] Wolff AC, Hammond ME, Schwartz JN, Hagerty KL, Allred DC, et al (2007) American Society of Clinical Oncology/College of American Pathologists guideline recommendations for human epidermal growth factor receptor 2 testing in breast cancer. Arch Pathol Lab Med 131: 18–43. 19548375 10.5858/2007-131-18-ASOCCO

[17] Singletary SE, Connolly JL (2006) Breast cancer staging: working with the sixth edition of the AJCC Cancer Staging Manual. CA Cancer J Clin 56: 37–47; quiz 50–31. 10.3322/canjclin.56.1.37 16449185

[18] Assi HA, Khoury KE, Dbouk H, Khalil LE, Mouhieddine TH, et al (2013) Epidemiology and prognosis of breast cancer in young women. J Thorac Dis 5: S2–8. 23819024 10.3978/j.issn.2072-1439.2013.05.24 PMC3695538

[19] Anders CK, Hsu DS, Broadwater G, Acharya CR, Foekens JA, et al (2008) Young age at diagnosis correlates with worse prognosis and defines a subset of breast cancers with shared patterns of gene expression. J Clin Oncol 26: 3324–3330. 18612148 10.1200/JCO.2007.14.2471

[20] Azim HA Jr, Michiels S, Bedard PL, Singhal SK, Criscitiello C, et al (2012) Elucidating prognosis and biology of breast cancer arising in young women using gene expression profiling. Clin Cancer Res 18: 1341–1351. 22261811 10.1158/1078-0432.CCR-11-2599

[21] Han M, Liu M, Wang Y, Chen X, Xu J, et al (2012) Antagonism of miR-21 reverses epithelial-mesenchymal transition and cancer stem cell phenotype through AKT/ERK1/2 inactivation by targeting PTEN. PLoS One 7: e39520. 22761812 10.1371/journal.pone.0039520 PMC3382593

[22] Zhu S, Si ML, Wu H, Mo YY (2007) MicroRNA-21 targets the tumor suppressor gene tropomyosin 1 (TPM1). J Biol Chem 282: 14328–14336. 17363372 10.1074/jbc.M611393200

[23] Zhu S, Wu H, Wu F, Nie D, Sheng S, et al (2008) MicroRNA-21 targets tumor suppressor genes in invasion and metastasis. Cell Res 18: 350–359. 18270520 10.1038/cr.2008.24

[24] Jiang S, Zhang HW, Lu MH, He XH, Li Y, et al (2010) MicroRNA-155 functions as an OncomiR in breast cancer by targeting the suppressor of cytokine signaling 1 gene. Cancer Res 70: 3119–3127. 20354188 10.1158/0008-5472.CAN-09-4250

[25] Kong W, He L, Coppola M, Guo J, Esposito NN, et al (2010) MicroRNA-155 regulates cell survival, growth, and chemosensitivity by targeting FOXO3a in breast cancer. J Biol Chem 285: 17869–17879. 20371610 10.1074/jbc.M110.101055 PMC2878550

[26] Cimino D, De Pitta C, Orso F, Zampini M, Casara S, et al (2013) miR148b is a major coordinator of breast cancer progression in a relapse-associated microRNA signature by targeting ITGA5, ROCK1, PIK3CA, NRAS, and CSF1. Faseb j 27: 1223–1235. 23233531 10.1096/fj.12-214692

[27] Chang H, Zhou X, Wang ZN, Song YX, Zhao F, et al (2012) Increased expression of miR-148b in ovarian carcinoma and its clinical significance. Mol Med Rep 5: 1277–1280. 22344713 10.3892/mmr.2012.794

[28] Ma L, Teruya-Feldstein J, Weinberg RA (2007) Tumour invasion and metastasis initiated by microRNA-10b in breast cancer. Nature 449: 682–688. 17898713 10.1038/nature06174

[29] Falkenberg N, Anastasov N, Rappl K, Braselmann H, Auer G, et al.. (2013) MiR-221/-222 differentiate prognostic groups in advanced breast cancers and influence cell invasion. Br J Cancer. 10.1038/bjc.2013.625 PMC3833215 24129242

[30] Zhao H, Shen J, Medico L, Wang D, Ambrosone CB, et al (2010) A pilot study of circulating miRNAs as potential biomarkers of early stage breast cancer. PLoS One 5: e13735. 21060830 10.1371/journal.pone.0013735 PMC2966402

[31] Lu Z, Ye Y, Jiao D, Qiao J, Cui S, et al (2012) miR-155 and miR-31 are differentially expressed in breast cancer patients and are correlated with the estrogen receptor and progesterone receptor status. Oncol Lett 4: 1027–1032. 23162645 10.3892/ol.2012.841 PMC3499613

[32] Song CG, Wu XY, Fu FM, Han ZH, Wang C, et al (2012) [Correlation of miR-155 on formalin-fixed paraffin embedded tissues with invasiveness and prognosis of breast cancer]. Zhonghua Wai Ke Za Zhi 50: 1011–1014. 23302487

[33] Qian B, Katsaros D, Lu L, Preti M, Durando A, et al (2009) High miR-21 expression in breast cancer associated with poor disease-free survival in early stage disease and high TGF-beta1. Breast Cancer Res Treat 117: 131–140. 18932017 10.1007/s10549-008-0219-7

[34] Yan LX, Huang XF, Shao Q, Huang MY, Deng L, et al (2008) MicroRNA miR-21 overexpression in human breast cancer is associated with advanced clinical stage, lymph node metastasis and patient poor prognosis. Rna 14: 2348–2360. 18812439 10.1261/rna.1034808 PMC2578865

[35] Li CI, Beaber EF, Tang MT, Porter PL, Daling JR, et al (2013) Reproductive factors and risk of estrogen receptor positive, triple-negative, and HER2-neu overexpressing breast cancer among women 20–44 years of age. Breast Cancer Res Treat 137: 579–587. 23224237 10.1007/s10549-012-2365-1 PMC3547981

